# 197 Impact of De-implementing Gowning and Gloving Requirements for COVID-19 on Environmental Waste Creation

**DOI:** 10.1017/ash.2026.10585

**Published:** 2026-06-23

**Authors:** Alexia Foy-Crowder, Rachel Samples, Joseph Kohn, Andrew Jolly, Sangita Dash, Majdi Al-Hasan, Caroline Jozefczyk, Pam Bailey

**Affiliations:** 1 Prisma Health; 2 Prisma Health - Midlands; 3 Prisma Health/University of South Carolina; 4 University of South Carolina School of Medicine; 5 Christiana Care Health System

## Abstract

**Background:** In February 2024, Prisma Health, a 17-hospital healthcare system, changed the reflex urine culture criteria to ≥10 white blood cells (WBC) and rejecting cultures if ≥10 epithelial squamous cells in urinalysis (previously included leukocyte esterase and nitrites). In May 2025, we added verbiage to the Epic orderable emphasizing the criteria. **Methods:** Using the EMR, lists of urinalyses were generated before the reflex criteria change (August 2023, n=229), after (March 2024, n=102), and an additional data pull June/July 2025 to identify patients who had urinalyses done, and to further identify those who had rejected cultures based on the reflex criteria. A subset for pre/post were reviewed, and all patients with rejected cultures were reviewed. Their charts were reviewed and relevant data captured in RedCap databases. **Results:** Comparing pre-change (n=229) and after (n=102), there was no difference in the two groups for bacteriuria, leukocyte esterase, WBC, nitrites, and epithelial squamous cells (p=0.31). Antimicrobial prescribing was similar between the groups (p=0.17). Out of 24,978 urinalyses with microscopy in June/July 2025, 291 patients had urinalyses that were rejected for cultures (0.012%). Of the 291 patients, 5 were male, 3 nonbinary, and the rest female. Fifty-eight were pregnant persons. Mean body mass index was 32 mg/m2. The top five most common chief complaints were gastrointestinal (n=86), genitourinary (n=46), obstetric (n=41), gynecologic (n=17), musculoskeletal (n=17). For clinician reasoning from note review, most were ordered for suspected urinary tract infection or urinary symptoms (n=134), no rationale documented (n=52), obstetric workup (n=51), metabolic workup (n=16) or sepsis workup (n=16). Ten patients returned to the ED subsequently, but none required additional workup related to their rejected culture. **Conclusions:** There were no differences between the characteristics of patients between the reflex culture changes. Very few cultures ultimately were rejected due to skin contamination, emphasizing that rejecting samples for culture is a viable option to improve diagnostic stewardship. The predominance of female and obese patients with contaminated urinalyses emphasizes additional attention to urine collection techniques. Fifty eight obstetric patients were ordered routine urinalyses, not following the orderset obstetric patients excluded from reflex culturing; providers need to be educated to ensure they follow pathways in place. Improved documentation about symptoms and different uses for urinalysis is is key for interpretation of this test.

